# Smoking and Obstructive Sleep Apnea: Is There An Association between These Cardiometabolic Risk Factors?—Gender Analysis

**DOI:** 10.3390/medicina57111137

**Published:** 2021-10-20

**Authors:** Despoina Ioannidou, George Kalamaras, Serafeim-Chrysovalantis Kotoulas, Athanasia Pataka

**Affiliations:** 1 Department of Respiratory Medicine, Papageorgiou General Hospital, 564 29 Thessaloniki, Greece; depioannidou@gmail.com; 2 Respiratory Failure Unit, G. Papanikolaou Hospital, School of Medicine, Aristotle University of Thessaloniki, 57010 Thessaloniki, Greece; kalamaras.giorgos@gmail.com (G.K.); akiskotoulas@hotmail.com (S.-C.K.)

**Keywords:** sleep apnea, smoking, gender differences, cardiovascular, hypertension

## Abstract

*Background and Objectives*: Studies have tried to establish a relationship between Obstructive Sleep Apnea syndrome (OSA) and smoking but data still remain controversial. We aimed: 1. To evaluate the relationship between smoking and OSA; 2. To explore potential differences according to gender, and 3. To analyze the prevalence of cardiovascular disease (CVD) co-morbidities according to gender and smoking status. *Materials and Methods*: This retrospective study included 3791 (70.6% males) adult patients who visited a Sleep Clinic. All participants underwent nocturnal polysomnography. Daytime somnolence and insomnia were assessed by using the Epworth Sleepiness Scale (ESS) and the Athens Insomnia Scale (AIS). Ever-smokers completed the Fagerstrom Test for Nicotine Dependence (FTND). *Results*: OSA was confirmed in 72.1% of participants with 62.2% suffering from moderate-to-severe disease. The number of cigarettes/day, Pack/Years, and FTND were significantly higher in patients with more severe OSA. The prevalence of current smokers was higher in those without OSA or with mild disease, whereas the prevalence of former smokers was higher in moderate and severe OSA. In univariate analysis, current smokers were found to be 1.2 times more likely to have OSA compared with never and former smokers combined and former smokers 1.49 times more likely compared with never smokers. In the multiple regression analysis, after adjusting for BMI, gender, age and number of alcoholic drinks per week, smoking was not found to be significantly associated with OSA. In gender stratified multivariate analyses, no significant associations were observed. CVD co-morbidities were more frequent in more severe OSA. Hypertension, coronary disease and diabetes were more prevalent in former smokers with AHI ≥ 15, compared with current smokers, especially in men. *Conclusions*: Even if an independent effect of smoking on OSA was not found, the number of cigarettes/day, Pack/Years, and FTND were higher in patients with more severe OSA with more prevalent CVD co-morbidities.

## 1. Introduction

Obstructive sleep apnea syndrome (OSA) is the most common sleep-related breathing disorder characterized by snoring, repeated episodes of airflow cessation, hypoxemia during sleep, and daytime hypersomnolence [1,2]. It is estimated that 2% to 14% among community screened patients and 21% to 90% among patients referred for sleep evaluation suffer from OSA [1]. Based on recent data, the prevalence of OSA ranges from 13% to 33% in men and from 6% to 19% in women, respectively [3]. Women may be particularly vulnerable to OSA during life events like pregnancy and menopause as reproductive hormone levels change [4,5]. Additionally, females appear to have a different OSA clinical presentation compared with males with lower apnea hypopnea index (AHI). Women report less snoring and episodes of respiratory events than men, while insomnia, fatigue, headaches and mood changes are reported more frequently. As a consequence of this different clinical profile, OSA may be under-diagnosed in females [5].

Many studies have tried to establish the relationship between OSA and cigarette smoking, showing a higher prevalence of smoking among OSA patients [6,7]. Additionally, there is evidence that smoking may be a risk factor for sleep apnea or snoring [8,9,10,11,12,13]. Smokers present decreased sleep quality with difficulties in initiation and maintenance of sleep [14,15,16,17]. Smoking induces upper airway chronic inflammation contributing to OSA symptoms [18,19]. Active, passive, as well as former smoking has been associated with snoring [11]. However, the data still remain controversial, failing to conclusively establish a clinically significant association between these two entities. Despite the conflicting evidence, it is reported that smoking has an impact on OSA through various mechanisms including changes of sleep architecture, of upper airway neuromuscular function, of arousal mechanisms and of enhanced upper airway inflammation [13]. Additionally, untreated OSA has been related to smoking addiction [20,21]. Nevertheless, further research is needed to clarify the effect of each disorder on the other. Gender may also influence outcomes, as especially in females, OSA has been found to independently increase the risk for coronary heart disease and type 2 diabetes mellitus [22]. On the other hand, some studies suggest a higher risk of cardiovascular disease (CVD) co-morbidity [23] in men suffering from OSA.

CVD are frequent comorbidities in OSA patients [18]. OSA is associated with an increased incidence of arterial hypertension (HTN), stroke, heart failure, atrial fibrillation, and coronary artery disease (CAD) [24]. OSA prevalence ranges between 30% to 83% in patients with HTN, between 12–55% in those with heart failure and 38–65% in patients with CAD [24]. It is reported that OSA patients, especially those with the severe form of the syndrome, exhibit higher all-cause and CVD mortality [24]. OSA is also independently associated with metabolic syndrome and insulin resistance that are related with an increased risk for incident CVD events [25,26]. In any case, the pathogenesis of CVD in OSA patients is multifactorial including various well-characterized mechanisms such as intermittent hypoxia, oxidative stress, sympathetic activation, and endothelial dysfunction [24].

Cigarette smoking is also a major risk factor of CVD and remains the leading cause of preventable mortality worldwide. The all-cause mortality rate among smokers is almost three times than that of never smokers [13], whereas the adverse effects of smoking on CVD risk have been demonstrated to be greater in women than in men of the general population [27,28]. The purpose of this study was to evaluate the relationship between smoking and OSA and to explore potential differences according to gender. We also aimed to analyze and compare the prevalence of cardiovascular co-morbidities of OSA patients according to gender and smoking status.

## 2. Materials and Methods

We conducted a retrospective cohort study, including all adult patients who visited the Sleep Clinic of Respiratory Failure Unit of Aristotle University of Thessaloniki due to symptoms suggestive of OSA over the years 2015–2020. Data regarding patients’ demographic and clinical characteristics, co-morbidities and smoking habits were recorded. CVD (arterial hypertension, history of myocardial infarction, coronary artery disease, stroke) and diabetes mellitus type 2 were self-reported by the patients. Patients with unclear smoking history, those already receiving treatment for OSA, or suffering from other sleep disorders apart from OSA were excluded. The Local Ethics Committee has approved the protocol (No965/290618), and all participants have given their consent.

Daytime somnolence was assessed with the use of the Epworth Sleepiness Scale (ESS) [29]. An ESS score greater than 10 points was considered as excessive daytime sleepiness. The 8-item Athens Insomnia Scale (AIS) was used to evaluate the severity of insomnia [30]. An AIS cut-off score of ≥6 was used to establish the diagnosis of insomnia problems. All participants underwent nocturnal polysomnography (Embletta^®^ GOLD, Portable Sleep System, Embla, Broomfield, CO, USA) to confirm the diagnosis of OSA. Sleep studies were manually scored according to the American Academy of Sleep Medicine (AASM) criteria [31]. Cases were stratified according to the severity of OSA determined by the Apnea Hypopnea Index (AHI) as mild with AHI 5 to 15/h, as moderate with AHI greater than 15 to 30/h and as severe with AHI greater than 30/h.

Patients with an active smoking history > 2 years were considered current smokers, and those with no smoking history as non-smokers. Smokers were divided into two groups: current and former smokers. Former smokers referred to smokers who quit smoking for at least six months. Smoking history was quantified in number of pack years (P/Ys) as (packs smoked per day) × (years as a smoker). Active and former smokers completed the Fagerstrom Test for Nicotine Dependence (FTND) [32].

### Statistical Analysis

SPSS for Windows 20.0 software (SPSS Inc., Chicago, IL, USA) was used for the statistical analysis. Descriptive statistics for continuous variables and for categorical data were given in terms of average and standard deviation and frequency and percentage, respectively. Chi-square test was used to compare categorical data. A comparison of quantitative data was done using unpaired *t*-test and, where appropriate, one-way ANOVA with post hoc Bonferroni comparison. Univariate analyses and multivariate logistic regression analyses were performed to calculate the odds ratio (OR) and 95% confidence intervals (95% CI) for smoking status and the risk of OSA and for the relationship of smoking history with CVD co-morbidities. All multivariate logistic regression analyses controlled for a number of known risk factors that included sex, age, BMI and number of alcoholic drinks per week. Current and former smokers were compared separately with never smokers for the likelihood of developing OSA. Current smokers were also compared with former and never smokers combined, for the likelihood of developing OSA. All significance tests were two-sided, and a 0.05 significance level was used.

## 3. Results

A total number of 3791 participants, males (70.6%) and females (29.4%), were recruited in the study. After evaluation by a sleep study, 520 (13.7%) of the participants had AHI < 5, 534 (14.1%) suffered from mild disease, 918 (24.2%) from moderate and 1819 (48%) from severe. The mean ESS score was higher in patients with moderate to severe OSA as well as insomnia symptoms (Table 1). There was a marked male predominance, whereas OSA patients were older with higher body mass index (BMI). Of all the participants, 30.5% were current smokers, 32.9% former smokers and 32.9% never smokers. The percentage of smokers did not differ between patients with AHI ≥ 15 and those with AHI < 15. However, nicotine dependence, expressed with FTND, was higher in patients with AHI ≥ 15. The prevalence of obesity and all cardiovascular co-morbidities was higher in patients with AHI ≥ 15 when compared to AHI < 15 group (Table 1).

When the participants were compared according to OSA severity, significant differences were observed in age, BMI, AHI and Oxygen Desaturation Index (ODI) as expected (Table 2). In addition, the number of cigarettes/day, the P/Ys and FTND were significantly higher in patients with more severe OSA. Interestingly, the prevalence of current smokers was higher in those without OSA or with mild disease, whereas the prevalence of former smokers was higher in moderate and severe OSA. Furthermore, excessive daytime sleepiness (ESS) and insomnia symptoms (AIS) were increased according to severity of the disease.

In a separate analysis according to the smoking habits of the participants with OSA, former smokers were found to be older with a higher BMI and co-morbidities as hypertension, coronary heart disease and diabetes. Current smokers presented higher AHI and ODI, especially compared with non-smokers in the post hoc analysis. However, no significant differences were observed in ESS and AIS between groups (Table 3).

A significant but weak correlation was found between P/Ys and AHI (r = 0.12, *p* < 0.001), P/Ys and ODI (0.14, *p* < 0.001), P/Ys and ESS (r = 0.08, *p* < 0.001) and P/Ys and AIS (r = 0.04, *p* = 0.02). Additionally, a weak correlation was found between FTND and AHI (r = 0.09, 0.01), FTND and ODI (r = 0.08, *p* = 0.02) and FTND and AIS (r = 0.18, *p* < 0.001). In the analysis according to gender, for men the correlation remained similar. However, no significant correlations were found for women neither for P/Ys nor for FTND and AHI, ODI and AIS, respectively.

In a separate analysis of ever smoking patients with OSA (AHI ≥ 15) according to gender, a male predominance was observed (70.9% vs. 46.5%, *p* < 0.001). Interestingly, no significant difference in ESS score was observed, whereas women presented with more insomnia symptoms (*p* = 0.001) (Table 4).

Cigarettes/day, P/Ys and FTND were statistically significantly higher in men. HTN and diabetes were more prevalent in women but men presented more frequently CAD or a myocardial event. Comparing male and female OSA patients according to smoking status (current, former, and non-smokers), we found that current smokers were diagnosed at younger age and had higher BMI than former and never smokers in both genders. Smoking habit was associated with neither ESS nor OSA severity. As for co-morbidities, there were no statistically significant differences according to smoking status in women apart from a predominance of HTN in former smokers (*p* = 0.006); however, male former smokers presented all CVD co-morbidities (except stroke) and diabetes mellitus more frequently than active or never smokers (Table 5).

In the univariate analysis, smoking was significantly associated with OSA (*p* = 0.026) (Table 6). Current smokers were found 1.2 times more likely to have OSA compared with never and former smokers combined (95% CI 1.02–1.38). Univariate tests to estimate the odds of OSA for current vs. never smokers and former vs. never smokers were done using logistic regression. Interestingly, former smokers were 1.49 times more likely to have OSA compared with never smokers (95% CI 1.25–1.78). However, in the multiple regression analysis, after adjusting for BMI, gender, age and number of alcoholic drinks per week, smoking was not significantly associated with OSA (*p* = 0.76). Similarly, former smokers were not found more likely than never smokers to have OSA, in contrast to the univariate analysis where the result was statistically significant. In the subgroup gender analysis, former male smokers were 1.6 times more likely to present OSA, whereas current smokers were 1.2 times as likely to have OSA compared with never and former smokers combined. However, these results were not maintained after adjustment for confounding factors. In the female subgroup, no associations were statistically significant (Table 6).

Additionally we performed a multivariate analysis (adjusting for age, gender, smoking status, number of alcoholic drinks per week, AHI) on the relationship of smoking history with CVD co-morbidities that showed that smoking history was not so strongly associated with HTN (OR = 1.009, (95% CI 0.84–1.21), *p* = 0.9 for current smokers, OR = 0.968 (95% CI 0.8–1.16), *p* = 0.7 for former smokers) as female gender (OR = 1.5 (95% CI 1.28–1.77), *p* < 0.001), age (OR = 1.07 (1.07–1.08), *p* < 0.001) and AHI (OR = 1.01 (1.007–1.013), *p* < 0.001). Stroke was associated with current smoking (OR = 1.54 (0.93–2.55), *p* = 0.09) and age (OR = 1.05 (1.04–1.07), *p* < 0.001). CAD was strongly associated with current (1.98 (1.4–2.79), *p* < 0.001) and former smoking (OR = 2.49 (1.85–3.35), *p* < 0.001).

## 4. Discussion

In the current study, we found that the number of cigarettes/day, the P/Ys and nicotine dependence were significantly higher in patients with more severe OSA. The prevalence of current smokers was higher in those without OSA or with mild disease, whereas the prevalence of former smokers was higher in moderate and severe OSA. In univariate analysis, current smokers were found 1.2 times more likely to have OSA compared with never and former smokers combined and former smokers 1.49 times more likely compared with never smokers. However, in the multiple regression analysis, after adjusting for BMI, gender, age and number of alcoholic drinks per week, smoking was not found to be significantly associated with OSA. CVD co-morbidities were more frequent in more severe OSA. However, HTN was more strongly associated with gender and age than smoking status, whereas stroke and CAD were strongly associated with smoking. A higher prevalence of HTN was observed in patients with AHI ≥ 15 and especially those with more severe OSA. However, HTN, CAD, and diabetes were more prevalent in former smokers with AHI ≥ 15, compared with current smokers with AHI ≥ 15. In the separate analysis according to gender, a male predominance in smoking among OSA patient was observed, with male patients smoking more cigarettes, having higher nicotine dependence and more severe OSA than females. Daytime sleepiness did not differ between genders, but women complained more of insomnia. HTN and diabetes were more frequent in women but men presented more frequently CAD or an ischemic myocardial event. Finally, we observed that current smokers were diagnosed with OSA at a younger age compared with former and never smokers and presented higher AHI and oxygen desaturation Index (ODI).

The association between smoking and OSA is not currently well-established. Data from the Wisconsin Sleep Cohort Study report that active but not former smoking was associated with a greater possibility of developing moderate or severe OSA, even after adjustment of confounding factors especially in heavy smokers [21]. Similarly, a small case-control study in a referral population showed that the prevalence of current smoking in the group of OSA patients was higher than that of the non-OSA patients (35% vs. 18%) and that smoking was independently associated with OSA [8]. In addition, similarly with our findings, another study reported that heavy smokers suffered from more severe OSA and that smoking associated with earlier age of disease diagnosis [33]. A more recent study also revealed a significant effect of P/Ys, age and BMI on OSA severity. Increased smoking status was associated with increased OSA severity expressed by increased AHI and reduced oxygen saturation [34].

Possible smoking-associated mechanisms of OSA have been described through various studies. It was suggested that smoking alters the uvular mucosa of OSA patients, so that it becomes more thickened and edematous through CGRP-induced neurogenic inflammation. Longer exposure to smoking may result in a higher prevalence of moderate or severe OSA [19]. Nasal obstruction due to smoking-related chronic mucosal inflammation, as thickened epithelium, cellular hyperplasia, mucosal edema and damaged cilia function, was also considered to be a potential mechanism [35,36,37]. Other potential explanations of the smoking effects on OSA were suggested to be the nicotine-induced impairment of the neuromuscular protective reflexes of the upper airway [38]. Smoking-related respiratory effects, such as accelerated loss of lung function and increased rates of obstructive airway diseases [39] were also suggested to play a role in the development of OSA. Finally, the interactions among the stimulant effects of nicotine, of nicotine withdrawal and of the respiratory effects of smoking may result in sleep-disordered breathing. Older studies [40] demonstrated that the administration of nicotine gum prior to sleep resulted in a decreased number of obstructive and mixed apneas during the first 2 h of sleep, suggesting that nicotine may actually reduce sleep-disordered breathing. However, as nicotine blood levels decline and upper airway resistance increases during the night, AHI increased due to nicotine withdrawal or smoking-associated respiratory effects [40]. However, other studies using nicotine in the form of transdermal patches [41] and tooth patch [42] did not show any significant effects on respiratory events.

On the other hand, other studies suggest that there is no evidence of a causal relationship between OSA and cigarette smoking. In our study, smoking was not found to be significantly associated with OSA after adjusting for BMI, gender, age and number of alcoholic drinks per week, in both genders. However, the number of cigarettes/day, the P/Ys and nicotine dependence were significantly higher in patients with more severe OSA and both AHI and ODI were lower in non-smokers. In compliance with our results, a previous large cross-sectional study concluded that smoking was not an independent risk factor for OSA after adjusting for confounding variables (age, BMI, and gender). Patients with more severe OSA (AHI > 50) were found to be heavier smokers and conversely, heavy smokers presented a higher AHI than non-smokers [43]. Our findings are also in accordance with a recent study that reported no significant association between cigarette smoking and OSA after adjusting for gender, BMI and age [44]. This study also found that smokers presented higher AHI than in our results [44]. Furthermore, a large single-center retrospective observational study including 3613 OSA patients also reported that smokers with OSA had a higher AHI, and lower mean oxygenation during sleep [6]. In a like matter, in another retrospective analysis, no significant differences in the AHI were found when comparing current/former smokers with non-smokers. However, current/former smokers presented lower nocturnal mean oxygen saturation [45]. Opposite to our findings, these three latter studies [6,44,45] reported higher daytime sleepiness (ESS) in smokers that was attributed to the combined effects of nicotine on sleep architecture, on the upper airway and possibly to nocturnal hypoxia.

Additionally, older studies have also reported a significant decrease in nocturnal oxygen saturation among smokers, but no significant differences in AHI and ODI between smokers and non-smokers [46]. In another study, smoking was found to be associated with ODI and arousals but not with AHI. The effects were more pronounced in current than former smokers. Current smokers with more than 15 P/Ys presented higher Total Sleep Time at SaO2 <90% and higher arousal index. Further, former smokers with more than 15 P/Ys were found to have higher AHI and arousal index compared with those with less than 15 P/Ys [47]. In a more recent meta-analysis, OSA was related with the use of alcohol, without enough evidence to confirm its association with tobacco or caffeine. However, the level of evidence of this meta-analysis was low, so that the authors suggested a cautious interpretation of their results [48]. Similarly to our results, data from the Sleep Heart Health Study concluded that former smoking was associated with more severe OSA, although current smoking was not. An inverse association between AHI and current smoking was observed in both males and females of this study with current smokers been more likely to have lower AHI [49]. In addition, a large population study also found current smoking to be strongly but inversely associated with self-reported OSA diagnosis in both genders [50]. The most likely explanation for the discordant results of the aforementioned studies is the different populations examined. Studies like ours evaluate populations referred to a sleep clinic with the participants suffering more frequently from more severe OSA, being predominantly male and obese. On the other hand, studies evaluating population in the community setting include similar proportions of men and women with milder disease.

In our study, the majority of the ever-smoking patients were men who suffered from a more severe form of the disease in accordance with previously presented results [33]. Indeed, our results are expected, taking into account an OSA reported male:female ratio of 8–10:1 in clinical studies or approximately 2:1 or 3:1 in epidemiological studies [51]. Additionally, a cross-sectional study conducted in Greece, found that male sex was a strong independent determinant for current smoking [52]. The prevalence of smoking found in our study was 29 to 33%, similar to that of the general population in Greece (28–35%) [52,53], higher in males and OSA patients. Daytime sleepiness did not differ between genders. However, women complained more often of insomnia in accordance with the existing literature [5]. Whether different anthropometric measurements, possible hormonal changes or lifestyle factors influence our findings is under discussion. In any case, even if our current knowledge supports normal changes in sleep architecture according to sex, age, and ethnicity [4,5,54], the role of smoking status remains a potential field of future research.

Many studies have suggested a relationship between OSA and cardiovascular diseases [26,55]. In Greece, the prevalence of hypertension is almost 23% in men and 15% in women, with a lower prevalence of diabetes (around 5%) in both genders [56]. The prevalence of CVD co-morbidities was higher in our OSA population compared with the general population. HTN is the most common CVD co-morbidity in patients suffering from OSA [24,25,26,57]. On the other hand, smoking is one of the most important risk factors for HTN [58]. We found a significantly higher prevalence of HTN in patients with AHI ≥ 15 and especially those with more severe OSA. Paradoxically, we also found that HTN was less frequent in smokers with AHI ≥ 15, compared with former smokers and also current smokers with AHI ≥ 15. Our results are in line with the observations of Bielicki et al. [6] that also showed that the proportion of non-smokers with OSA with HTN was higher than that of ever-smokers. Contrary to our findings, Shao et al. found a significant increase of HTN risk in current/former smoking patients with OSA [45]. This variation could be related to the differences of the populations studied. In the study of Bielicki et al. [6], as in our study, smokers were younger than non-smokers and former smokers, and this could have had an impact on the results, as HTN incidence increases with age [59]. In our analysis HTN was more strongly associated with gender and age than smoking status. Additionally, our study, due to its retrospective design, did not assess the duration of smoking cessation in former smokers, a factor that could also affect HTN.

Regarding the separate gender analysis, the prevalence of HTN and diabetes mellitus among female smoker patients and especially former smokers was higher than in male. This was expected, considering that female patients were older and more obese. On the other hand, among male OSA patients that smoked, CAD and myocardial infarction were more frequent. Notably, a recent large epidemiological study of Greek OSA patients showed that female sex was an independent significant predictor of prevalent CVD (arterial hypertension, coronary heart disease, stroke, and heart failure), especially in younger and non-obese OSA patients [60]. Indeed, important gender-based differences have been described in the association between OSA and CVD [22,27,28,61,62]. Furthermore, there is evidence supporting a significant role of OSA as a contributing factor to incident CVD and cardiac remodeling in women, suggesting that these associations are stronger than in middle aged to elderly male individuals without prevalent CVD [63]. Our findings though can be expected, since the majority of male patients in our cohort suffered from more severe disease and smoked more cigarettes/day and had more P/Ys than female patients. Actually, previous studies have shown that moderate to severe OSA was more strongly associated with CVD risk than gender [60]. Whether there is a potential modifying role of smoking on the gender-related cardiovascular effect of the syndrome needs to be further evaluated.

The present study has certain limitations. Firstly, patients were enrolled based on a referral to the sleep clinic, a factor that potentially limits the ability to generalize our findings to other populations. Additionally, the retrospective design of the study precludes causal inferences, and the self-reported data may lead to underestimation of smoking or CVD prevalence. However, these limitations are partly counterbalanced by our relatively large sample.

## 5. Conclusions

In conclusion, even if we failed to prove an independent effect of smoking habit on OSA, we showed that the number of cigarettes/day, the P/Ys, and nicotine dependence were significantly higher in patients with more severe OSA. Current smokers were diagnosed with OSA at a younger age compared with former and never smokers and presented higher AHI and ODI. HTN, CAD, and diabetes were more prevalent in former smokers with AHI ≥ 15, compared with current smokers with AHI ≥ 15. A higher prevalence of HTN was observed in patients with AHI ≥ 15 and especially those with more severe OSA. To our knowledge, this is the first epidemiological study examining a large cohort of Greek subjects and investigating the effect of smoking habits on OSA and the potential sex differences.

## Figures and Tables

**Table 1:** Clinical characteristics of the population and comparison between subjects with AHI ≥ 15 vs. AHI < 15.

	All Subjects (*n* = 3791)	AHI ≥ 15 Subjects (*n* = 2733)	AHI < 15 Subjects (*n* = 1058)	*p*
Age, years	57.2 ± 13.6	58.9 ± 12.6	53.1 ± 15.04	<0.001
BMI, kg/m^2^	32.1 ± 9.2	33.4 ± 9.6	28.6 ± 7.2	<0.001
Male gender, *n* (%)	2678 (70.6)	2007 (73.4)	671 (63.4)	<0.001
Smokers, *n* (%)	1158 (30.5)	802 (29.3)	356 (32.9)	0.09
FTND	4.9 ± 2.8	5.1 ± 2.6	4.49 ± 2.9	0.007
ESS	9.6 ± 4.5	10.12 ± 4.5	8.35 ± 4.3	<0.001
AIS	8.9 ± 5.5	9.2 ± 5.48	8.01 ± 5.4	<0.001
Obesity ^a^, *n* (%)	2343 (61.8)	1941 (71)	402 (38)	<0.001
Arterial hypertension, *n* (%)	1530 (40.4)	1217 (44.5)	313 (29.6)	<0.001
Coronary heart disease	400 (10.6)	332 (12.1)	68 (6.4)	<0.001
Myocardial infarction, *n* (%)	96 (2.5)	82 (3)	14 (1.3)	<0.001
Stroke, *n* (%)	107 (2.8)	22 (2.1)	85 (3.1)	0.4
Diabetes mellitus, *n* (%)	488 (12.9)	393 (14.4)	95 (9)	<0.001

OSA: obstructive sleep apnea syndrome; BMI: body mass index; FTND: Fagerstrom Test for Nicotine Dependence; ESS: Epworth sleepiness scale; AIS: Athens Insomnia Scale; ^a^: BMI > 30.

**Table 2:** Characteristics of the participants according to the severity of OSA.

	No OSA (*Ν* = 520)	Mild OSA (*Ν* = 534)	Moderate OSA (*Ν* = 918)	Severe OSA (*Ν* = 1819)	*p*
Age (years)	49.4 ± 15.1	56.7 ± 14	59 ± 12.7	58.8 ± 12.5	<0.001
BMI (kg/m^2^)	27.7 ± 7	29.6 ± 7.2	31.3 ± 8.9	34.4 ± 9.7	<0.001
Cigarettes/day	20.1 ± 15	22.6 ± 15.5	24.7 ± 16.3	27.7 ± 18	<0.001
Pack/years	13.5 ± 26.01	19.36 ± 26.1	22.84 ± 30.45	25.96 ± 32.86	<0.001
Current smokers (%)	178 (34.2)	176 (32.9)	269 (29.3)	535 (29)	0.12
Former smokers (%)	118 (22.7)	170 (31.8)	318 (34.6)	642 (35.2)	0.01
FTND	4.19 ± 2.93	4.85 ± 2.7	4.9 ± 2.67	5.23 ± 2.85	0.008
AHI (events/h)	2.4 ± 1.2	9 ± 2.9	22.8 ± 3.8	53.3 ± 19.1	<0.001
ODI (events/h)	2.9 ± 3.5	9.1 ± 4.2	22.9 ± 7.7	52.4 ± 20.6	<0.001
ESS	8.4 ± 4.2	8.2 ± 4.3	9.3 ± 4.4	10.5 ± 4.6	<0.001
AIS	8.2 ± 5.5	7.7 ± 5.2	8.8 ± 5.4	9.4 ± 5.4	<0.001
Arterial hypertension, *n* (%)	118 (22.7)	195 (36.5)	378 (41.2)	839 (46.1)	<0.001
Coronary artery disease, *n* (%)	23 (4.4)	45 (8.4)	113 (12.3)	219 (12)	<0.001
Diabetes mellitus, *n* (%)	43 (8.26)	67 (12.5)	140 (15.2)	348 (19.1)	<0.001
Myocardial infarction, *n* (%)	2 (0.4)	13 (2)	27 (2.9)	60 (3.3)	0.48
Stroke, *n* (%)	9 (1.7)	13 (2.4)	27 (2.9)	58 (3.2)	0.74

OSA: obstructive sleep apnea syndrome; BMI: body mass index; AHI = Apnea-Hypopnea Index; ODI = Oxygen Desaturation Index; FTND: Fagerstrom Test for Nicotine Dependence; ESS: Epworth sleepiness scale; AIS: Athens Insomnia Scale.

**Table 3:** Characteristics of the participants with OSA according to their smoking habits.

	Smokers (*Ν* = 802, 29.3%)	Former Smokers (*Ν* = 959, 35.1%)	Non-Smokers (*N* = 886, 32.4%)	*p*
Age (years)	54.4 ± 11	62.1 ± 11	59.5 ± 13	<0.001
BMI (kg/m^2^)	34.2 ± 8.2	34.2 ± 8.6	33.1 ± 9.5	0.01
Neck circumference, cm	43 ± 6.8	43.3 ± 6.5	41.6 ± 5.8	<0.001
Waist circumference, cm	116 ± 18.1	118 ± 15.6	114 ± 16.5	0.006
AHI (events/h)	44.2 ± 22.4	43.1 ± 21.1	41.7 ± 20	0.048
ODI (events/h)	43.4 ± 22.7	42.09 ± 22.4	40.9 ± 21.08	0.047
ESS	9.9 ± 4.67	10.26 ± 4.5	10.1 ± 4.6	0.42
AIS	9.24 ± 5.2	9.1 ± 5.7	9.37 ± 5.47	0.66
Arterial hypertension, *n* (%)	307 (38.3)	472 (49.2)	424 (47.8)	<0.001
Coronary artery disease, *n* (%)	75 (9.3)	189 (19.7)	64 (7.2)	<0.001
Diabetes mellitus, *n* (%)	121 (15.1)	217 (22.6)	146 (16.5)	<0.001
Myocardial infarction, *n* (%)	24 (3)	51 (5.3)	12 (1.35)	0.9
Stroke, *n* (%)	27 (3.4)	31 (3.2)	26 (2.9)	0.8

OSA: obstructive sleep apnea syndrome; BMI: body mass index; AHI: Apnea-Hypopnea Index; ODI: Oxygen Desaturation Index; ESS: Epworth sleepiness scale; AIS: Athens Insomnia Scale.

**Table 4:** Characteristics of ever-smokers OSA patients (AHI ≥ 15) according to gender.

	Males (*N* = 1423)	Females (*N* = 338)	*p*
Age (years)	58.5 ± 12.5	58.9 ± 10	0.01
BMI, (kg/m^2^)	33.8 ± 7.7	36 ± 10.7	0.02
Waist circumference, cm	130 ± 17	110 ± 18	0.002
Neck circumference, cm	42.9 ± 6.5	38.8 ± 7.6	<0.001
ESS	10.14 ± 4.5	10.1 ± 4.6	0.5
AIS	8.7 ± 5.3	10.8 ± 5.6	0.001
AHI (event/h)	45.4 ± 21.6	40.3 ± 22	0.007
ODI (event/h)	43.5 ± 21.9	41.2 ± 25	0.2
FTND	5.26 ± 2.6	4.5 ± 2.8	0.03
Cigarettes/day	24.6 ± 14.4	17.1 ± 9.9	<0.001
Packs/year	38.9 ± 27	26 ± 19.1	<0.001
Arterial hypertension, *n* (%)	606 (42.6)	173 (51)	0.005
Coronary artery disease, *n* (%)	240 (16.8)	24 (7.1)	<0.001
Diabetes mellitus, *n* (%)	200 (14)	71 (21)	0.002
Myocardial infarction, *n* (%)	64 (4.49)	6 (1.77)	0.045
Stroke, *n* (%)	27 (3)	8 (2.8)	0.5

OSA: obstructive sleep apnea syndrome; BMI: body mass index; ESS: Epworth sleepiness scale; AIS: Athens insomnia scale; AHI: apnea hypopnea index; ODI: oxygen desaturation index; FTND: fagerstrom test for nicotine dependence.

**Table 5:** Comparison of smokers, former smokers and non-smokers with OSA (AHI ≥ 15) according to gender.

	Men with OSA (*N* = 2007)				Women with OSA (*N* = 726)			
	Current Smokers (*n* = 632)	Former Smokers (*n* = 791)	Non-Smokers (*n* = 584)	*p*	Current Smokers (*n* = 170)	Former Smokers (*n* = 168)	Non-Smokers (*n* = 388)	*p*
Age (years)	54.4 ± 11.1	62.1 ± 11.8	59.5 ± 13.5	<0.001	56.3 ± 9.6	61.4 ± 9.8	64.3 ± 11.8	<0.001
BMI, (kg/m^2^)	33.9 ± 7.4	33.7 ± 8.1	32.6 ± 8.6	0.015	35.5 ± 10.6	34.8 ± 10.7	33.8 ± 10.5	0.019
ESS	10 ± 4.6	9.9 ± 4.6	10.2 ± 4.5	0.42	10.1 ± 4.7	10 ± 4.5	9.5 ± 4.4	0.24
AHI (event/h)	45.3 ± 22.2	43.7 ± 21	43.1 ± 20	0.17	40.1 ± 22.7	40.4 ± 21.3	39.7 ± 18.6	0.9
ODI (event/h)	43.9 ± 21.8	43.2 ± 22.07	41.8 ± 21.4	0.23	39.7 ± 20.4	41.4 ± 25.9	41.1 ± 24.1	0.65
Arterial hypertension, *n* (%)	232 (36.7)	374 (47.2)	211 (36.1)	<0.001	75 (44.1)	98 (58.3)	213 (54.8)	0.006
Coronary artery disease, *n* (%)	67 (10.6)	174 (21.9)	38 (6.5)	<0.001	9 (5.3)	15 (8.9)	27 (7)	0.43
Stroke, *n* (%)	22 (3.4)	26 (3.2)	14 (2.3)	0.76	5 (2.9)	5 (3)	12 (3.1)	0.86
Diabetes mellitus, *n* (%)	63 (9.9)	137 (17.3)	59 (10.1)	<0.001	38 (22)	33 (19.6)	38 (9.8)	0.218
Myocardial infarction, *n* (%)	19 (3.6)	45 (6.06)	9 (1.9)	0.001	2 (1.17)	4 (2.3)	3 (0.77)	0.17

OSA: obstructive sleep apnea syndrome; BMI: body mass index; ESS: Epworth sleepiness scale; AHI: apnea hypopnea index; ODI: oxygen desaturation index.

**Table 6:** Risk of Obstructive Sleep Apnea (OSA) associated with current smoking and former smoking estimated by unadjusted and adjusted Odds Ratio (OR) and 95% Confidence Intervals (CI).

Total OSA Sample	Univariate				Multivariate *			
	OSA OR (95% CI)		*p*		OSA OR (95% CI)		*p*	
Current vs. never or former smoker	1.2 (1.02–1.38)		0.026		1.037 (0.87–1.22)		0.67	
Current vs. never smoker	1.01 (0.85–1.2)		0.84		0.98 (0.81–1.19)		0.88	
Former vs. never smoker	1.49 (1.25–1.78)		<0.001		1.08 (0.89–1.3)		0.39	
**Subgroup Analysis by Gender**	**Males**				**Females**			
	**Univariate**		Multivariate **		**Univariate**		Multivariate **	
	OSA (95% CI)	*p*	OSA (95% CI)	*p*	OSA (95% CI)	*p*	OSA (95% CI)	*p*
Current vs. never or former smoker	1.23 (1.02–1.48)	0.02	1.06 (0.8–1.3)	0.5	1.2 (0.9–1.6)	0.1	0.96 (0.7–1.3)	0.8
Current vs. never smoker	1.05 (0.8–1.3)	0.6	1.0 (0.7–1.2)	0.2	1.25 (0.9–1.6)	0.1	0.96 (0.7–1.3)	0.8
Former vs. never smoker	1.6 (1.2–2)	<0.001	1.2 (0.9–1.5)	0.1	1.01 (0.7–1.3)	0.9	1.04 (0.7–1.4)	0.7

* Odds ratios have been adjusted for age, sex, body mass index and number of alcoholic drinks per week. ** Odds ratios have been adjusted for age, body mass index and number of alcoholic drinks per week.

## Data Availability

The data presented in this study are available on request from the corresponding author.
